# Ruthenium(II) and Platinum(II) Complexes with Biologically Active Aminoflavone Ligands Exhibit In Vitro Anticancer Activity

**DOI:** 10.3390/ijms22147568

**Published:** 2021-07-15

**Authors:** Małgorzata Fabijańska, Maria M. Kasprzak, Justyn Ochocki

**Affiliations:** Departament of Bioinorganic Chemistry, Medical University of Lodz, Muszyńskiego 1, 90-151 Lodz, Poland; kasprzak.maria@interia.eu (M.M.K.); justyn.ochocki@umed.lodz.pl (J.O.)

**Keywords:** medicinal chemistry, platinum compounds, ruthenium compounds, biologically active compounds, cytotoxicity evaluation, apoptosis

## Abstract

Continuing our studies on the mechanisms underlying the cytotoxicity of potential drugs, we have described several aspects of the in vitro anticancer activity of ruthenium(II) and platinum(II) complexes with bioactive, synthetic aminoflavone ligands. We examined the mechanism of proapoptotic activity of *cis*-dichlorobis(3-imino-2-methoxyflavanone)ruthenium(II), *cis*-dichlorobis(3-imino-2-ethoxyflavanone)ruthenium(II), and *trans*-dichlorobis(3-aminoflavone)platinum(II). Cisplatin was used as a reference compound. The cytotoxicity was investigated by MTT assay. The mechanism of proapoptotic activity of the tested compounds was investigated by evaluation of caspase-8 activity, cytometric analysis of annexin-V positive cells, and mitochondrial potential loss measurement. The results showed that ruthenium compounds break partially or completely the cisplatin resistance by activating the caspase 8-dependent apoptosis pathway and loss of mitochondrial membrane potential. Platinum compounds also have a cytostatic effect, but their action requires more exposure time. Potential mechanisms underlying drug resistance in the two pairs of cancer cell lines were investigated: total glutathione content, P-glycoprotein activity, and differences in the activity of DNA repair induced by nucleotide excision. Results showed that cisplatin-resistant cells have elevated glutathione levels relative to sensitive cells. Moreover, they indicated the mechanisms enabling cells to avoid apoptosis caused by DNA damage. Pg-P activity has no effect on the development of cisplatin resistance in the cell lines described.

## 1. Introduction

Drug resistance remains one of the biggest challenges in cancer therapy. Intense research has been carried out during the past decades and several mechanisms that are responsible for the cisplatin-resistant phenotype of tumor cells have been described. Cisplatin resistance may occur at four different stages: during the drug circulation through the bloodstream, during the drug influx and efflux through the cell membrane, during its presence in cytoplasm, and finally after DNA binding. As cisplatin has been found to be the major therapeutic options in some clinical situations, the development of innovative strategies that overcome drug resistance constitutes a goal with important clinical implications [1].

Over the last 40 years, large numbers of platinum-metal compounds with anticancer activity have been synthesized, such as mono- and multi-nuclear platinum complexes, trans-platinum complexes, Pt(IV) complexes, and other synthesis strategies. Interestingly, the outcomes of clinical trials of these complexes resembled the classical platinum complexes and none of the non-classical platinum complexes has been approved for clinical application. This phenomenon has encouraged many scientists to be engaged in designing novel platinum complexes [2]. Therefore, it is necessary to introduce new antitumor platinum complexes, with a modern structure, that may have anticancer potentials. The main approaches in the development of novel platinum-based complexes are the following: changing ammonia/leaving groups, conjugation with bioactive/active molecules, conjugation with stereochemistry, coordination saturation, introduction of axial ligands, and merging two or more platinum coordination spheres. In brief, the platinum drug development field occupies a significant position and is still being broadened [3,4].

Among the transition metal complexes, ruthenium complexes have received attention in many aspects. A number of Ru-based anticancer agents have been developed to date, yet none of them are in clinical use as anticancer drugs. Successful entries to clinical trials of NAMI-A [5], KP1019 [6], NKP1339 [7], and TLD1443 [8,9], together with many reports on the promising in vitro and in vivo activities of other types of Ru complexes, have caused Ru-based chemotherapeutics to be seen as a major area in anticancer drug research [10,11,12].

An interesting methodology used for designing new bioactive metal compounds with anticancer properties is based on preparing coordination compounds bearing ligands that present some biological activity. In this way, flavonoids, which are largely used as pharmaceutical agents due to their beneficial properties, often have been used as ligands to synthesize active complexes. Compounds based on the flavone skeleton also exhibit multiple biological activities. To be precise, many flavonoids have been shown to have antioxidant, antimicrobial, and even anticancer effects [13]. Nevertheless, the biological activity of the flavonoids is not limited to antioxidant properties. Some flavonoids may be inhibitors of important enzymes (e.g., topoisomerases and aromatase) [14,15] or mutagens and/or DNA intercalators; the interaction of some flavonoids with enzymes and DNA may underlie their cytostatic properties [16].

**Figure 1:**
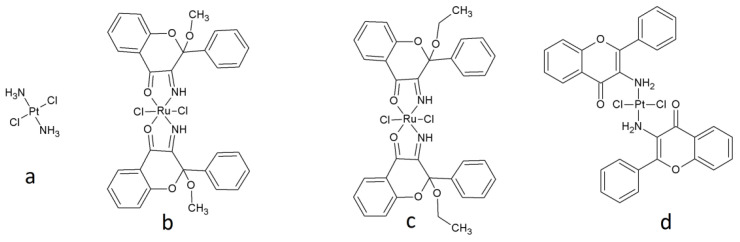
Structures of the compounds: (**a**)—*cis*-diamminedichloroplatinum (II) (cisplatin, CDDP) **1**; (**b**)—*cis*-dichlorobis (3-imino-2-methoxyflavanone)ruthenium (II) (134) **2**; (**c**)—*cis*-dichlorobis(3-imino-2-ethoxyflavanone)ruthenium(II) (138) **3**; (**d**)—*trans*-dichlorobis(3-aminoflavone)platinum(II) (TCAP) **4**.

Our study has three main experimental parts: (1) we evaluated the properties of the biological material (the cisplatin-resistant cell lines compared to cisplatin-sensitive cell line); (2) assessed the cytotoxicity of the ruthenium(II) and platinum(II) compounds with flavonoid bioligands previously obtained by our group (cis-dichlorobis(3-imino-2-methoxyflavanone)ruthenium(II) (134) **2** [17], cis-dichlorobis(3-imino-2-ethoxyflavanone)ruthenium(II) (138) **3** [17], and trans-dichlorobis(3-aminoflavone)platinum(II) (TCAP) **4** [18], with cis-diaminadichloroplatinum(II) (CDDP or cisplatin) 1—an anticancer drug—used as a reference compound) (see Figure 1), also studying the mechanism of action of the tested compounds on the cisplatin-resistant and cisplatin-sensitive lines; and (3) compared these results to the compound activity against normal cells (human lymphocytes).

The potential mechanisms underlying drug resistance in the tested cell lines, i.e., total glutathione content, P-glycoprotein (Pg-P multidrug resistance factor) activity, and differences in DNA repair activity by nucleotide excision (NER), were examined. 

The ability of the tested compounds to overcome cisplatin resistance in tumor cells in vitro with 72 h exposure, as well as the mechanisms activated by them to induce cell death (apoptosis) in cells of a cisplatin-sensitive ovarian cancer line (A2780) and its cisplatin-resistant derivative (A2780cis) were investigated. Furthermore, cancer cell lines with different sensitivity to cisplatin were used in this study, including two pairs of cancer cell lines: human ovarian cancer A2780 (cisplatin-sensitive cells) and A2780cis (cisplatin-resistant), and human Toledo lymphoma (cisplatin-sensitive) and Toledo-cis (cisplatin-resistant). 

## 2. Results

### 2.1. Mechanism of Resistance to Cisplatin in Cancer Cell Lines

Cells develop resistance to platinum agents by establishing a complicated self-protection system to escape toxic metal compounds. Primarily, under-expression of membrane transporters [19] or overexpression of drug efflux pumps [20] contribute to the reduced levels of platinum accumulation in the cell. Differences in the expression of proteins involved in protection against or predisposition towards DNA damage, such as heat shock [21] and ribosomal proteins [22], are also implicated. Furthermore, epigenetic changes, such as in DNA methylation [23], structural changes in chromatin [24], and histone modifications [25], as well as involvement of diverse transcription factors [26] and post-transcriptional gene-regulating microRNA [27], may contribute to the development of resistance [28]. In this paper, the following selected aspects underlying drug resistance in the A2780 and A2780cis lines were investigated: total glutathione content, P-glycoprotein (Pg-P multidrug resistance factor) activity, and differences in DNA repair activity by nucleotide excision.

#### 2.1.1. Concentration of Total Glutathione 

Glutathione (γ-L-glutamyl-L-cysteinylglycine) is a thiol tripeptide that plays a crucial role in cellular redox equilibrium and disposal of toxic substances. Due to the presence of a sulfhydride group, it has a high potential to bind and inactive toxic metal ions, including metal base drugs, e.g., cisplatin. Therefore, elevated level of glutathione is commonly found in cisplatin-resistant cancer cells [1], as a crucial pre-target resistance mechanism. It also can be a cause of cellular cross resistance to other cytostatic agents, especially metal-based ones, and those acting by oxidative stress in cancer cells. 

To determine whether a higher glutathione concentration was present in the drug-resistant cell lines, compared to their sensitive parent lines, we measured total glutathione in the sensitive-resistant line pairs, namely, A2780 vs. A2780cis and Toledo vs. Toledo-cis.

**Figure 2:**
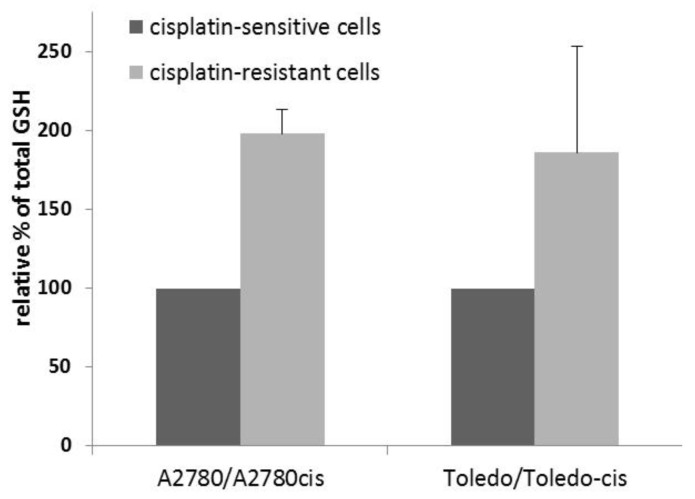
Relative total glutathione content in cells with different sensitivity to cisplatin; a colorimetric assay. The absorbance determining the glutathione concentration in cells of the sensitive lines was taken as 100% and the corresponding value for resistant lines was calculated in relation to it. Bars show the mean ± SD values from three separate measurements.

The results showed an approximately two-fold increase in glutathione concentration in resistant cells, when compared to sensitive cells (see Figure 2). Therefore, glutathione plays a significant role in the resistance to cisplatin in A2780cis and Toledo-cis cells.

#### 2.1.2. Activity of Glycoprotein P

Glycoprotein P (P-gP) is one of the ATPase transmembrane transporters that remove metabolites and toxic chemicals out of the cellular environment [29,30]. It is encoded by the MDR1 gene and plays a crucial role in multidrug resistance in cancer cells [31]. Overexpression of P-gp causes enhanced efflux of therapeutic agents from target cells, which allows us to classify it as a pre-target mechanism of resistance. The effect can usually be reversed by inhibitors of P-gp., e.g., verapamil, which is clinically applied to sensitize cancer cells to chemotherapy [32]. Rhodamine 123 (R-123) is a fluorescent dye that is also a substrate of P-gp. Therefore, it is a useful tool for the determination of P-gp activity in viable cells, by monitoring changes in the R-123 concentration in the cells in time, with or without the presence of P-gp inhibitors [33].

**Figure 3:**
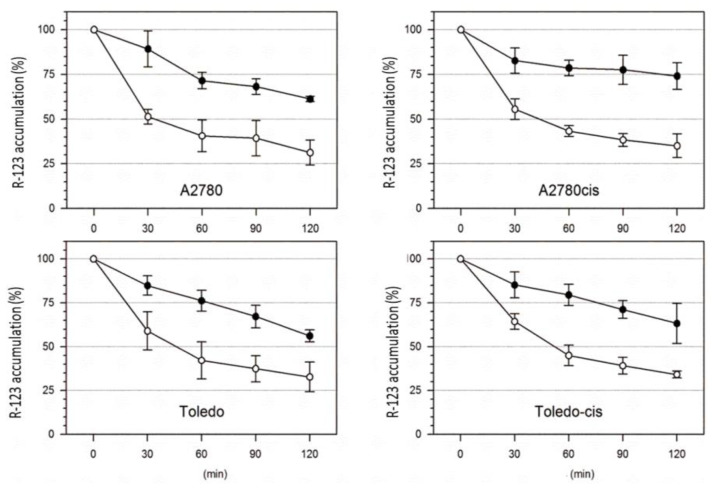
Kinetics of rhodamine 123 removal from cisplatin-resistant and -sensitive ovarian and Toledo cells. Cells were incubated in culture medium with 2 mol/L rhodamine for 2 h at 37 °C, and then washed and further incubated in a culture medium without rhodamine in the absence (○) or presence (●) of 10 mol/L of verapamil. Results shown are representative data of 4–6 individual studies.

Two pairs of cisplatin-sensitive and -resistant cell lines (A2780 vs. A2780cis and Toledo vs. Toledo-cis) were tested towards the differences in R-123 accumulation and efflux rates with or without verapamil. A higher rate of R-123 efflux in drug-resistant cells over the sensitive ones could be attributed to the higher activity of P-gp, and the effect should be reversed by verapamil. Our results showed no significant difference in P-gp activity between sensitive and resistant cells. The rate of accumulation and efflux of R-123 was similar in both cell line pairs (see Figure 3), and the process was similarly affected by verapamil. Therefore, it can be concluded that the resistance to cisplatin in A2780cis and Toledo-cis cells does not depend on a higher activity of P-gp.

#### 2.1.3. Efficacy of DNA Nucleotide Excision Repair 

The cytotoxic action of platinum compounds is mainly due to causing DNA damage. Cisplatin forms adducts with DNA, by binding N7 atoms in purine rings. Those adducts are removed by the cellular nucleotide excision repair (NER) system. The more effective NER is in a cell, the more efficient the cell can be in removing DNA–platinum adducts. This on-target mechanism of resistance can contribute to failures of platinum-based anticancer therapy [1,34]. Nucleotide excision repair is also the main mechanism coping with UV-induced DNA damage, namely, pyridine dimers. Therefore, genetic impairments in the NER system cause extreme sensitivity to sunlight in affected individuals, which is known as xeroderma pigmentosum [35]. The common way of DNA repair after damage caused by cisplatin and UV radiation can be used in the laboratory in assessing NER efficacy in cisplatin-resistant cancer cells in vitro. Damage to DNA after the exposure of the cells to cisplatin is hard to compare in different cell lines and requires a long time (several hours) to occur and can be affected by many factors, such as the drug transport mechanisms, glutathione-based inactivation, etc. Irradiation with UV is a quick, direct, and reproducible [36] method of inducing DNA damage in cultured cells, and the extent of the damage does not depend on intracellular pre-target resistance factors.

The nucleotide excision repair of DNA is executed in several steps: (i) recognition of the lesion; (ii) removal of the lesion and the immediate proximity in the affected DNA strand; and (iii) synthesis of the correct nucleotide sequence based on the intact DNA strand. The second step of NER generates DNA strand breaks, which can be observed by single-cell gel electrophoresis or comet assay. The measurement of the extent and kinetics of formation and subsequent repair of DNA strand breaks can be used as a tool to evaluate the cellular NER response to DNA-damaging stimuli [37,38,39].

We applied the comet assay to assess DNA repair after the exposure to UV-C irradiation in two pairs of cisplatin-sensitive and -resistant cell lines (A2780 vs. A2780cis and Toledo vs. Toledo-cis) to find out whether a higher NER capacity could be one of the underlying mechanisms of the resistance to cisplatin in A2780cis and Toledo-cis cells, compared to their parental lines.

**Figure 4:**
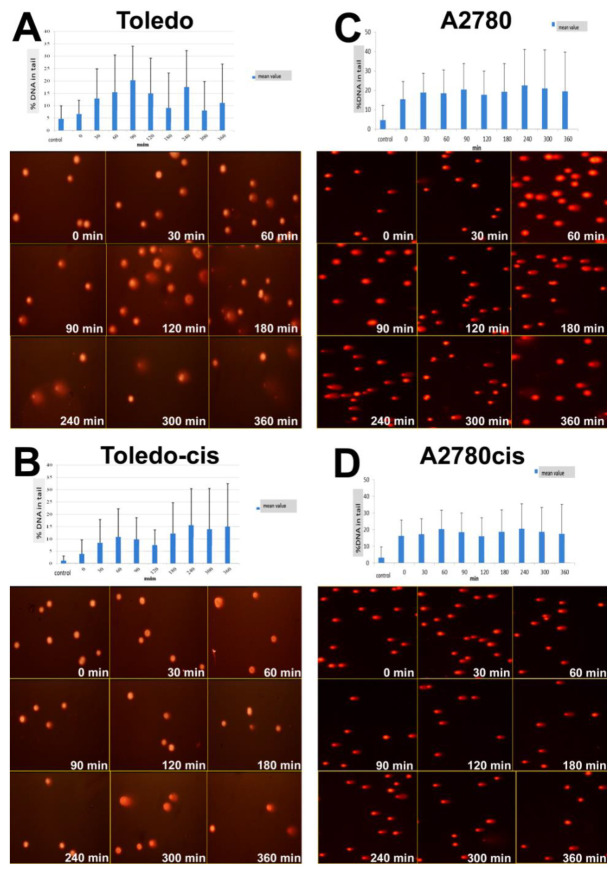
Efficacy of the DNA nucleotide excision repair—data are expressed as mean values of % DNA in the tail as a function of time after UV exposure in cisplatin-sensitive Toledo (**A**) and cisplatin-resistant Toledo-cis (**B**), and cisplatin-sensitive A2780 (**C**) and cisplatin-resistant A2780cis (**D**) cancer cell lines.

We found no significant difference between A2780 and A2780cis cells regarding the kinetics of DNA repair after UV exposure (see Figure 4C,D). However, under the conditions applied, the cell populations were not uniform in their DNA processing pattern. After 3 h, one could observe a subpopulation of the cells with a decreasing level of DNA strand break, and the second one with an increasing level of the damage, which indicated that they entered an apoptosis/necrosis pathway.

Toledo cells appeared to be much more sensitive to UV radiation than A2780 (see Figure 4A,B). The vast majority of the cell population exposed to UV apparently underwent apoptosis. However, Toledo-cis cells appeared to be much less influenced by the irradiation than ovarian carcinoma cells. The majority of the cells were very weakly influenced; there were no apparent patterns of creating and removing DNA breaks or the comet pattern characteristic of apoptotic nuclei. It is probable that Toledo-cis cells have evolved some kind of molecular by-pass, allowing them to overcome the repair-inducing or lethal effects of the UV irradiation; or, the onset of apoptosis is more delayed in them, beyond the tested 6 h post-exposure. Anyway, the low response of the cells to UV rays is probably connected with higher resistance of Toledo-cis to cisplatin, when compared to the Toledo cell line.

### 2.2. Cellular Viability Assay—MTT Test

The present study used a panel of four human cancer cell lines with human lymphocytes. The results have been partially published (IC_50_ for 134 (**2**), 138 (**3**), and cisplatin) in our previous paper [40]. For the sake of comparison, they are reposted along with the results for the TCAP(**4**) compound (Table 1).

A2780 (human ovarian cancer) and Toledo (B-lymphocyte human lymphoma) cells were very sensitive to cisplatin, with an IC_50_ value well below 1 µM. Cisplatin-resistant sublines were also used, with an IC_50_ value about an order of magnitude higher than those of the sensitive parent cell lines. Cisplatin-resistant subline A2780cis had an IC_50_ value of 16.8 µM and Toledo-cis, obtained in our laboratory, had an IC_50_ value of 8.3 µM. 

TCAP(**4**) revealed significant cytotoxicity towards all tested cell lines, with IC_50_ values in the 6.5–15.1 µM range (Table 1). However, it has much lower toxicity towards all the cell models when compared to ruthenium compounds **2** and **3**. This may seem surprising, as the platinum compounds are considered more cytotoxic than ruthenium compounds in general [41]. For TCAP(**4**), basically, no significant differences were observed between the sensitive (A2780 and Toledo cells) and their cisplatin-resistant sublines (A2780cis and Toledo-cis). We supposed that TCAP(**4**) has the ability to retain cytotoxic activity against cisplatin-resistant cell lines, which could be explained as different mechanisms of activity. Interestingly, TCAP(**4**) almost completely overcame cisplatin resistance in Toledo-cis cells. We previously found that ruthenium compounds **2** and **3** partially overcome cisplatin resistance in A2780cis and Toledo-cis cells (its toxicity in cisplatin-resistant cells is the same as in the sensitive cells—the resistance factor RF, expressed as IC_50_ resistant cells/IC_50_ sensitive cells—equals 1). The resistance factor for cisplatin in A2780cis/A2780 is 84. For the compound TCAP(**4**), it is 2.3. In case of Toledo-cis/Toledo cells, the resistance factor is about 17 for cisplatin and 1.4 for compound **4**. Therefore, while cisplatin is more toxic to almost all the cell lines than TCAP(**4**), the toxicity ratio of the resistant/sensitive cells is smaller in case of TCAP(**4**). Interestingly, compounds 134 (**2**), 138 (**4**) and TCAP(**4**) similarly overcome cisplatin resistance in ovarian cancer lines (RF = 1.8; 0.8; 2.3 respectively). This very desirable feature of the new compounds could be connected with a different mechanism of action from that of cisplatin (RF = 84).

Cisplatin was very toxic to normal lymphocytes, which were found to respond similarly to the sensitive cell lines. Compounds 134 (**2**) and 138 (**3**) are also less toxic to normal lymphocytes than cisplatin, which is a favorable property in the context of testing potential anticancer agents. Particularly noteworthy is the fact that TCAP was 60-times less toxic for normal lymphocytes in comparison to cisplatin, while compounds 134 (**2**) and 138 (**3**) were found to be 3 and 7 times less active, respectively. The low toxicity of compound 4 towards human lymphocytes is undoubtedly a desirable feature for the prevention of potential drug side-effects. It should also be noted that the free ligand 3-aminoflavone was not cytotoxic at <100 µM. 

### 2.3. Proapoptotic Activity of Tested Compounds

Apoptosis (programmed cell death) is a desired way in which cancer cells should respond to treatment. The efficacy and extent of the process can be evaluated by means of many indicators and at various points of apoptosis. A cell that undergoes programmed death initiates a cascade of biochemical changes, such as cell membrane modifications, activation of enzymes (e.g., caspases), loss of mitochondrial membrane polarization, fragmentation of nuclear DNA, cell shrinkage, and decomposition into apoptotic bodies. Any drugs that help induce cancer cells to enter the apoptosis pathway are therefore highly desirable for anticancer therapy. Our studies showed how apoptosis was induced by the tested compounds in human ovarian cancer cells sensitive to cisplatin (A2780) and resistant to cisplatin (A2780cis).

#### 2.3.1. Caspase 8 Activity Assay

We assessed the activation of caspase-8 in A2780 and A2780cis cells after their exposure to Ru(II) and Pt(II) compounds. Low activity of caspase-8 can indicate low induction of the “extrinsic” receptor apoptotic pathway, which can contribute to higher resistance of the cells to many cytostatic drugs [42]. The aim of this experiment was to find out whether the tested compounds activated the caspase-8 dependent apoptotic pathway, as well as whether there were any differences in its level between the drug-sensitive and -resistant cells.

**Figure 5:**
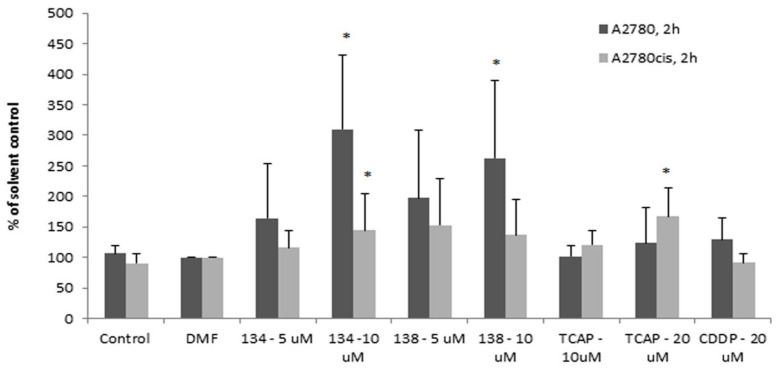

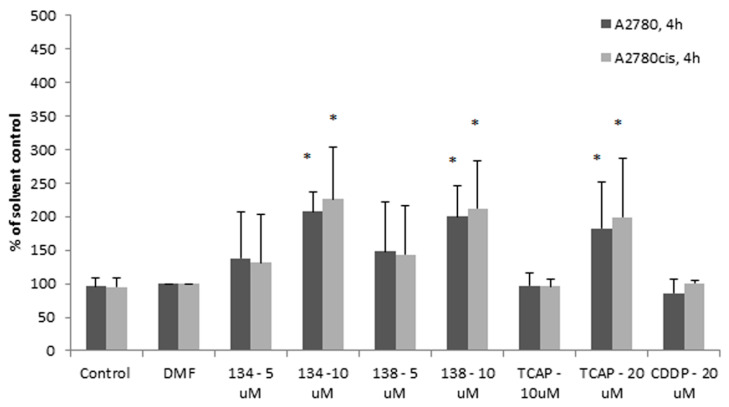
Caspase 8 activity in ovarian cancer cells with different sensitivity to cisplatin, measured by the colorimetric method, relative to the solvent control. Results shown are representative data of at least three individual studies (* *p* < 0.05).

The results showed that after 2 h, caspase-8 is activated by the Ru(II) compounds earlier and more efficiently in the sensitive A2780 cells than in the resistant A2780 cells (see Figure 5). Pt(II) compounds were less able to induce apoptosis under the same conditions, up to 4 h of exposure. This suggested a different mechanism of activity of the Pt(II) compounds vs. Ru(II) compounds. Apart from this difference, the delayed activation of caspase-8 in A2780cis cells may indicate that the impairment in the execution of the extrinsic apoptotic pathway can contribute to the resistance of the A2780cis cell line to cisplatin.

#### 2.3.2. Annexin V Apoptosis Assay

**Figure 6:**
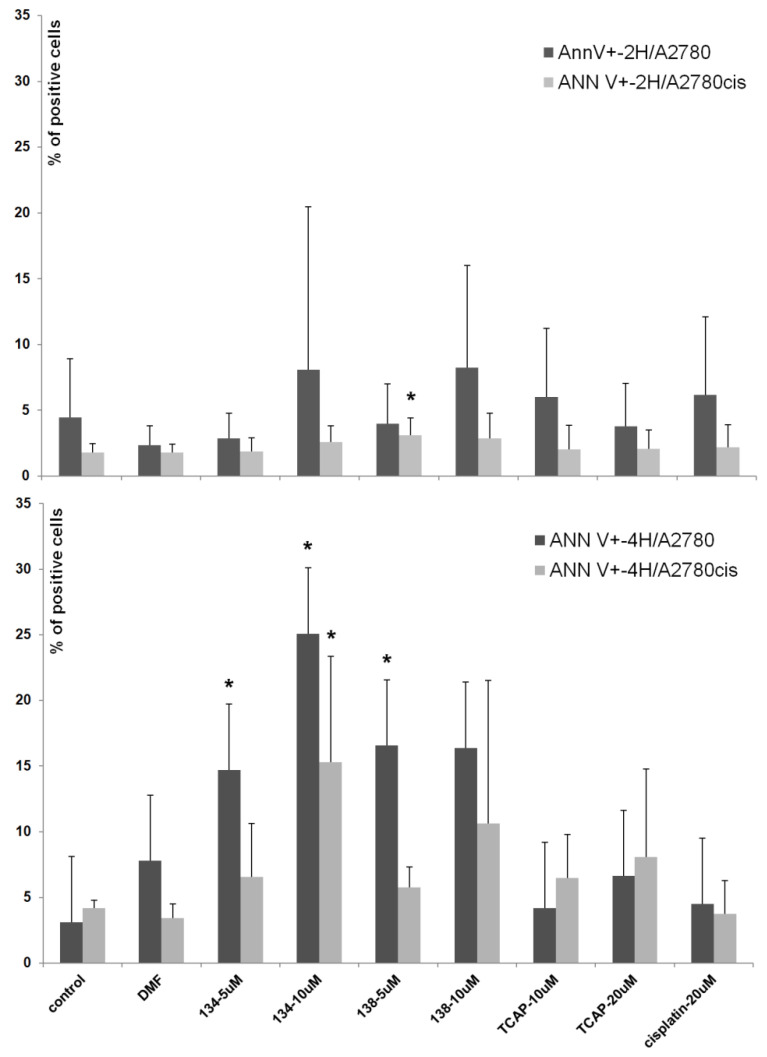
Flow cytometric analysis of annexin-V-positive cells. Percentage of apoptotic cells in A2780 and A2780cis cell cultures after 2 h and 4 h incubation with the tested compounds. Results shown are representative data of at least three individual studies (* *p* < 0.05).

Annexin-V (Ann-V) is a protein that has a high affinity to the phosphatidylserine present on an apoptotic cell’s membrane. When annexin-V is conjugated with a fluorescent dye (e.g., fluorescein), it can be used to detect apoptotic cells by means of spectrofluorometric flow cytometry. An additional stain, propidium iodide (PI), which marks only the already dead cells, can be used to distinguish between early apoptotic (Ann-V+, PI-), late apoptotic (Ann-V+, PI+), necrotic (Ann-V-, PI+), and living (Ann-V-, PI-) cells. In this study we assessed the apoptotic-inducing potential of Ru(II) and Pt(II) compounds in ovarian cancer cell lines A2780 (cisplatin sensitive) and A2780cis (cisplatin resistant). The Ru(II) compounds caused a significant dose- and time-dependent increase in the percentage of Ann-V positive cells of both lines, when compared to the control (untreated cells). The Pt(II) compounds, cisplatin and TCAP, did not cause similar changes under the same conditions (see Figure 6). This shows that the Pt(II) compounds induce cell death after a longer time or/and at higher concentrations than Ru(II)compounds.

#### 2.3.3. Mitochondrial Potential Loss Measurement

**Figure 7:**
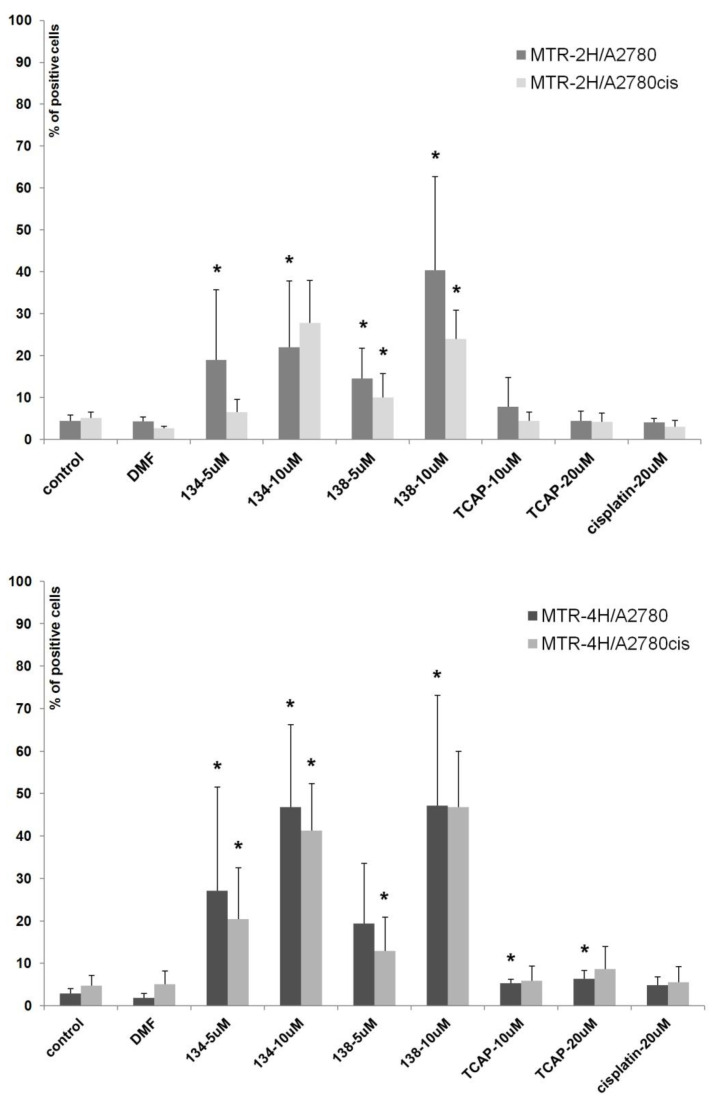
Flow cytometric analysis of A2780 and A2780cis cells with low mitochondrial transmembrane potential (MTR positive) after 2 h and 4 h incubation with the tested compounds. Results shown are representative data of at least three individual studies (* *p* < 0.05).

We assessed the influence of Pt(II) and Ru(II) compounds on mitochondria function in cells A2780 and A2780cis. Loss of mitochondrial membrane potential is a crucial hallmark of apoptosis [43]. The experiment showed that Ru(II) compounds induce the loss of mitochondrial membrane potential in a dose-dependent manner (see Figure 7). After 4 h of exposure, the percentage of A2780 and A2780cis cells with mitochondrial impairment was nine times greater than in control samples. TCAP and cisplatin under the same conditions had much lower influence on mitochondrial membrane potential. The results suggest that Ru(II) compounds induce apoptosis in a shorter time and a different manner than the tested Pt(II) compounds.

The main mechanism of Pt(II) compound activity depends on the interaction with DNA, causing crosslinks that hamper DNA replication and transcription. Apoptosis is executed soon after the DNA repair systems find the damage and send the signal to initiate the programmed death pathway. The process takes on average several hours to begin. The Ru(II) compounds induced apoptosis in a shorter time than Pt(II) compounds, which indicated a more direct way of activity, e.g., the caspase-8-dependent receptor pathway and mitochondrial (intrinsic) pathway, triggered probably by oxidative stress [44].

### 2.4. Interaction of Ru(II) and Pt(II) Compounds with Topoisomerases

Topoisomerases I and II are the enzymes that relieve torsional strain in supercoiled DNA (scDNA) during transcription and replication. Inhibition of the enzymes impairs cell division and expression of genetic information, thus leading to cell death. For this reason, the enzymes are targets for many cytostatic drugs. Topoisomerase I (Topo1) relaxes DNA by introducing one DNA strand incision (the so called “nick”), and after the strand unwinding, re-ligating the incision. There are Topo1 inhibitors among the antineoplastic agents, e.g., the camptothecin derivatives irinotecan and topotecan. Topoisomerase II (Topo2) causes a double-strand break in DNA and re-ligates it after unwinding. Cytostatic drugs that inhibit Topo2 are, e.g., etoposide and anthracyclines. Topoisomerase inhibitors are usually divided into two subclasses, depending on their mechanism of action: catalytic inhibitors and poisons. Catalytic inhibitors block the catalytic activity of the enzyme, while the poisons stabilize the topoisomerase–DNA complex. The Topo-inhibiting cytostatic drugs are in fact topoisomerase poisons. There are studies that have found topoisomerase inhibitors among natural flavonoids, e.g., genistein [45], luteolin [14], myricetin, and fisetin [46], as well as among ruthenium compounds [47,48]. 

**Figure 8:**
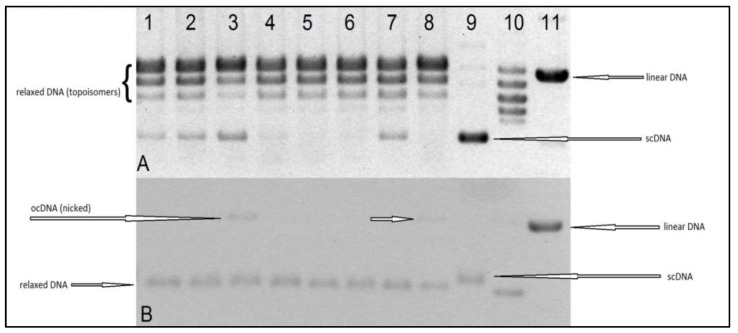
Electrophoregrams in 1% agarose gel of supercoiled (sc) plasmid DNA digested with topoisomerase I (topo1), without or in the presence of the indicated substances: 1—DNA + topo1 (enzyme control); 2—1% DMSO (solvent control), 3—100 µM camptothecin (positive control); 4—134 10 µM; 5—138 10 µM, 6—TCAP 10 µM; 7—TCAP 20 µM; 8—100 µM camptothecin (positive control); 9—scDNA; 10—relaxed DNA (topoisomers); 11—linear DNA. Image **A** shows the electrophoregram of an ethidium bromide-stained gel after electrophoresis, and image **B** shows the result of DNA separation in a gel with ethidium bromide.

**Figure 9:**
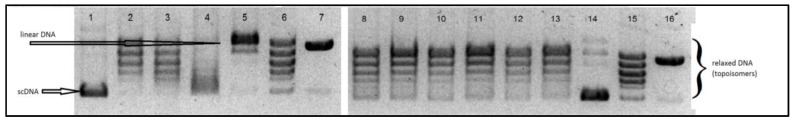
Electrophoregrams in 1% agarose gel of supercoiled (sc) plasmid DNA digested with topoisomerase II (topo2), without or in the presence of the indicated substances: 1—scDNA; 2–4—topo2 5, 10, and 15 units (enzyme activity control); 5—100 µM etoposide (positive control); 6—relaxed DNA (topoisomers); 7—linear DNA; 8—134 10 µM; 9—134 20 µM; 10—138 10 µM; 11—138 20 µM; 12—TCAP 10 µM; 13—TCAP 20 µM; 14—scDNA; 15—relaxed DNA (topoisomers); 16—linear DNA. Figure shows the electrophoregram of an ethidium bromide-stained gel after electrophoresis.

The aim of our experiments was to evaluate the interaction, in vitro, of the tested Ru(II) and Pt(II) compounds with human topoisomerases I and II, to assess their potential mechanism of cytostatic activity. The electrophoretogram of the scDNA treated with the enzyme and the Ru(II) and Pt(II) compounds shows the same band pattern as the negative control (scDNA digested with the enzyme only) (see Figure 8 and Figure 9). Therefore, we must conclude that the tested compounds do not inhibit topoisomerase I or II, nor lead to the formation of a stabilized DNA–Topo complex with open circular (topo1) or linear (topo2) DNA. For comparison, in the positive control, i.e., the sample of DNA treated with Topo1 and camptothecin (CPT), we observe the presence of open circular DNA, whereas the sample treated with Topo2 with etoposide shows the presence of linear DNA. Therefore, the cytotoxic activity of the tested metal compounds is most probably not based on inhibition of topoisomerases I and II.

## 3. Materials and Methods

### 3.1. Cell Culture 

#### 3.1.1. Continuous Cell Culture

Cell lines A2780 (human ovarian carcinoma, ECACC cat. No. 93112519), A2780cis (subline resistant to cisplatin, ECACC cat. No. 93112517), Toledo (human diffuse large cell lymphoma, ATCC CRL-2631TM), and Toledo-cis (cisplatin-resistant subline obtained from Toledo in our laboratory) were cultured in RPMI-1640 medium (Biological Industries, Kibbutz Beit-Haemek, Israel) supplemented with 10% heat-inactivated fetal bovine serum, 5 mM Hepes, and 50 µg/mL gentamycin. All cell lines were maintained in a humidified incubator, at 37 °C and 5% CO_2_. The cell lines A2780 and A2780cis were grown as monolayers, and cultures were detached with trypsin and renewed every 3–4 days. Toledo and Toledo-cis were grown in suspension; the cultures were renewed every 3–4 days. The Toledo cell line was kindly provided by Prof. Piotr Smolewski from the Department of Experimental Hematology, Medical University of Lodz, ul Ciołkowskiego 2, 93–510 Łódź, Poland, on the conditions of collaboration. The Toledo-cis cell line was obtained in our laboratory by exposing the original Toledo cells to cisplatin at increasing concentrations (0.25–3 µM in culture medium) for several months. 

#### 3.1.2. Human Blood Lymphocyte Culture

The buffy coat fraction of peripheral blood from healthy donors was purchased from the Regional Blood Bank in Lodz, Poland. Mononuclear blood cells were isolated using Lympho-Sep tubes (Biological Industries, Israel). Lymphocytes were cultured in RPMI-1640 medium (Biological Industries, Israel) supplemented with 10% heat-inactivated fetal bovine serum, phytohemaglutinin M solution (PHA-M, Biological Industries), 5 mM Hepes, and 50 µg/mL gentamycin.

### 3.2. Concentration of Total Glutathione 

The levels of glutathione (GSH) in the cells were determined by using a Glutathione Colorimetric Assay Kit (BioVision K261, Milpitas, CA, USA). Briefly, 1 × 10^6^ cells of each line were collected by centrifugation at 700× *g* for 5 min at 4 °C. The supernatant was removed. Cells were resuspended in 0.5 mL ice-cold PBS, transferred into 1.5 mL microcentrifuge tubes, and centrifuged at 700× *g* for 5 min at 4 °C. The supernatant was removed. Cells were lysed in 80 µL ice-cold Glutathione Buffer and incubated on ice for 10 min. Then, 20 µL of 5% SSA (sulfosalicylic acid) was added, mixed, and centrifuged at 8000× *g* for 10 min. The supernatant was transferred to a fresh tube and used for the glutathione assay. The GSH content of the sample was then assayed according to the instruction provided by the kit manufacturer, where the catalytic amounts of glutathione caused a continuous reduction in 5,5′-dithiobis-(2-nitrobenzoic) acid to 2-nitro-5-thiobenzoic acid (TNB). The oxidized GSH formed was recycled by the GSH reductase and NADPH. TNB was assayed calorimetrically. The results are shown as the mean of three independent experiments ± SD.

### 3.3. Activity of Glycoprotein P

A2780, A2780cis, Toledo, and Toledo-cis cells were seeded in 6-well test plates (2 × 10^5^ cells per 1 mL of medium, 3 mL of suspension per well). The procedure was conducted after 24 h. The cells were immersed in fresh, pre-warmed medium with addition of 10 or 20 μM rhodamine 123 dissolved in DMSO. Then verapamil was added to final concentrations of 0, 10, and 50 μM. The plates were incubated at 37 °C for 30 and 60 min. Then the cells were washed with ice-cold PBS and lysed in 2.5 mL of 0.5% TritonX-100 in distilled water. The fluorescence of the lysate was measured (SPECTROstarNano spectrophotometer, BMG Labtech, Ortenberg, Germany). The results are shown as the mean of three independent experiments ± SD.

The cells were immersed in fresh, pre-warmed medium with addition of 30 μM rhodamine 123 dissolved in DMSO and incubated at 37 °C for 45 min. Then the medium was replaced with the fresh one with addition of verapamil at concentrations of 0, 10, and 50 μM and incubated for 0, 30, and 60 min. Next, the cell monolayers were washed with ice-cold PBS and lysed in 2.5 mL of 0.5% TritonX-100 in distilled water. The fluorescence of the lysate was measured (SPECTROstarNano spectrophotometer, BMG Labtech, Ortenberg, Germany). The fluorescence of the control samples of each time (cells not treated with verapamil, time 0 min) was assumed as 1.00, as the reference for other samples. The results are shown as the mean of three independent experiments ± SD.

### 3.4. Efficacy of DNA Nucleotide Excision Repair

The test procedure was the same for all tested cell lines. A2780 and A2780cis cells were detached by trypsinization and carried into suspension (approximately 4–8 ×10^4^ cells per 1 mL of medium). The suspension was then transferred to a 12-well plate (1 mL of suspension to 1 well, the thickness of the suspension layer was approx. 2.6 mm). The plate was then exposed to UV light, from a 6W power source with a wavelength of λ = 254 nm, for 30 s at a distance of 14 cm. UV lamp power was 6 W = 6 Js^−1^; distance from UV lamp: d = 14 cm = 0.14 m; UV irradiance (Js^−1^m^−2^) = UV lamp power (Js^−1^)/4π d^2^; 6 Js^−1^/4 ∙ 3.14 (0.14m)^2^ = 24.4 J/s ∙ m^2^. UV dose (Jm^−2^) = UV intensity exposure time [36]. After UV exposure, the cell suspension was incubated for 30 min at 37 °C to allow the DNA repair process. The suspension was then centrifuged at 4–10 °C at 1500 rpm for 10 min and the culture medium was removed. Cells were resuspended in low melting point agarose (concentration of 1.2% agarose in PBS, a saline solution in phosphate buffer) and then applied to an agarose-coated primary slide. The base slide was covered with a coverslip and the whole thing was cooled. After this time, the coverslip was removed and the slides were placed in lysis buffer (2.5 M/L NaCl, 0.1 M/L EDTA, 0. 01 mol/L Tris, pH = 10) with 1% Triton X-100 and left until the next day at 4 °C. The preparations were then placed, for DNA denaturation, in an alkaline solution of pH > 13: 0.3 mol/L NaOH, 0.01 mol/L EDTA. Slides were incubated for 20 min and electrophoresed in the same buffer for 30 min, at 0.8 V/cm. After electrophoresis, the slides were neutralized in Tris-HCl buffer, pH = 7.5, washed with distilled water, and air-dried. Dry slides were stained with fluorescent dye (GelRed) and photographed with a camera connected to a fluorescence microscope. Comets were counted using CaspLab (version 1. 2. 3beta2) [49]. The percentage of DNA in the comet tail (a parameter that can be determined using CaspLab) was used to determine the DNA damage index, i.e., the sum of the single- and double-stranded DNA breaks [39]. The results are shown as the mean of at least three independent experiments ± SD.

### 3.5. Cellular Viability Assay—MTT Test 

The cancer cells were seeded in 24-well plates, with 1 mL of cell suspension per well, in the following densities: A2780—10^4^ cells/mL; A2780cis—1.5 × 10^4^ cells/mL; and Toledo and Toledo-cis—5 × 10^4^ cells/mL. Lymphocytes were seeded in 48-well plates, 0.5 mL of cell suspension per well, at −2 × 10^5^ cells/mL. The cultures were left overnight to adapt. The next day, the tested compounds were added, pre-dissolved in DMF, and then in a complete culture medium, at concentrations two-fold higher than the final ones. The resulting solutions were added to cell cultures to obtain the demanded concentration. Stock solutions of the compounds were freshly made before every experiment. The final concentration of the solvents in culture medium was 0.2% *v*/*v*. After 72 h, MTT (3-(4,5-dimethylthiazol-2-yl)-2,5-diphenyltetrazolium bromide) solution was added to PBS (final concentration 0.25 mg/mL in culture) and incubated at 37 °C for 2–3 h. Then the culture medium was removed, and the purple formazan crystals were dissolved in DMSO. The absorbance was measured at 540 nm on a Spectrostar Nano spectrophotometer with a microplate reader (BMG Labtech, Ortenberg, Germany). The experiments were performed at least in triplicate. The results were transformed to relative values, as the percentage of the solvent control (assumed as 100%) to subtract any toxic effect of DMF. 

### 3.6. Caspase 8 Activity Assay

The procedure was conducted according to the instructions provided by the kit manufacturer (*Caspase-8* Colorimetric Assay Kit, BioVision K113; Milpitas, CA, USA). A2780 and A2780cis cells were seeded in 6-well test plates (3 × 10^5^ cells per 1 mL of medium and 3 mL of suspension per well). The procedure was conducted after 24 h. The tested compounds were added to the cell cultures (one sample in each experiment was not treated with the compounds, for control) and incubated for 2 and 4 h. Then the cells were collected by means of trypsinization and centrifuged (10 min, 1500 rpm, 4 °C), rinsed with PBS + 0.01 M EDTA, and centrifuged again. The cell pellets were suspended in 0.055 mL of cell lysis buffer, stirred vigorously, incubated on ice for 10–20 min, and centrifuged (10 min, 12,000 rpm, 4 °C). In case of each assay, 0.050 mL of the supernatant (cell lysate) was collected and mixed with 0.050 mL of reaction buffer supplemented with dithiothreitol and IEDT-pNA (a substrate for caspase-8). The samples were then incubated at 37 °C, protected from light. Then the samples were diluted to the volume of 0.9 mL with a dilution buffer and absorbance was measured (λ = 405 nm). The results are showed as the mean of at least three independent experiments ± SD.

### 3.7. Annexin-V Apoptosis Assay

The apoptotic cells were identified by flow cytometry using the Annexin-V/FITC (BDBiosciences, San Diego, CA, USA) assay according to the manufacturer’s instructions. For detection of apoptosis and necrosis, FITC-labeled annexin-V combined with PI (propidium iodide) was used to mark the presence of phosphatidylserine (PS), which is displayed during apoptosis at the cell surface. PI only stains the nuclei of damaged cells with permeable plasma membranes. Briefly, the cells were incubated with tested compounds for 2 and 4 h. After incubation, the cells were washed twice with cold PBS and then resuspended in 100 μL of binding buffer, containing 2 μL of FITC conjugated annexin-V and 10 μg mL^−1^ of PI (Becton-Dickinson, San Jose, CA, USA). Then, the preparations were incubated at room temperature, protected from light, for 15 min. Fluorescence was measured immediately after staining by flow cytometry using FL1 (green, annexin-V) and FL3 (red, PI) standard fluorescent filters.

### 3.8. Mitochondrial Potential Loss Measurement

The loss of mitochondrial transmembrane potential (ΔΨm) occurs early during apoptosis and is often considered as a marker of apoptosis activated by the mitochondrial pathway. MitoTracker Red (Invitrogen, Waltham, MA, USA) dye was used as a probe for ΔΨm, which accumulates in the active mitochondria of living cells: 50 nM, 20 min incubation, at RT. The reduction of Δ Ψm was detected by flow cytometry as a decrease in red fluorescence of the dye in treated cells as compared to untreated cells.

### 3.9. Interaction of Compounds with Topoisomerases

The test procedure was carried out according to the manufacturer′s instructions (Topoisomerase I Drug Screening Kit(plasmid based) TopoGen/Topoisomerase II Drug Screening Kit (plasmid based) (TopoGen, Buena Vista, CO, USA). A reaction mixture with appropriate concentrations of water and buffer and 1–0.5 mL of DNA (plasmid) was prepared. Then, the tested compounds dissolved in DMSO were added. A solvent control test was also performed. Finally, an enzyme in the indicated amount was added to the mixture. The whole was incubated at 37 °C for 30 min. Then, the reaction was stopped by adding 2 μL of detergent-SDS (sodium dodecyl sulfate). Proteinase K solution (50 μg/mL) was then added and incubated at 37 °C for 15 min. After that time, a loading buffer (bromophenol blue and glycerol) was added to the samples and then applied to a 1% agarose gel. Electrophoresis was carried out in TAE (Tris Acetate-EDTA) buffer, which was obtained by mixing 40 mM Tris Acetate, 1 mM EDTA, and diluting with water. It was carried out for 4–5 h at 2 V/cm. For the Topo I assay, two types of gel were prepared: non-EB (Figure 8A) and EB gel (Figure 8B). Non-EB gels were stained with EB (0.5 ug/mL) for 15–30 min and then destained in water or buffer for 15 min prior to photodocumentation. EB gel was run in the presence of 0.5 ug/mL (in gel and running buffer), then destained with water for 15 min prior to photodocumentation. The difference is due to the fact that the ethidium bromide present in the gel binds DNA and gives the alternative possibility to distinguish different forms of DNA (linear, supercoiled, and nicked). Binding EtBr to DNA changes the charge, weight, conformation, and flexibility of the DNA molecule. Those two results (Figure 8A,B) are complementary and should be interpreted jointly. 

### 3.10. Data Analysis

The results are expressed as the mean ± SD. Statistical significance was assessed with a non-parametric Wilcoxon–Cox test; a *p* value less than 0.05 was considered as statistically significant. All statistical analyses were performed using StatSoft Statistica 10PL software.

## 4. Conclusions

The potential mechanisms underlying drug resistance in two pairs of cancer cell lines were investigated: total glutathione content, P-glycoprotein (Pg-P multidrug-resistance factor) activity, and differences in the activity of DNA repair induced by nucleotide excision. 

Higher level of glutathione is usually found in cisplatin-resistant cancer cells, as an essential pre-target resistance mechanism. It also can be a cause of cellular cross resistance to other cytostatic agents, principally metal-based compounds. Our results confirmed that cisplatin-resistant cells have elevated glutathione levels relative to sensitive cells. Moreover, they indicated the mechanisms enabling cells to avoid apoptosis caused by DNA damage. The results show that Toledo-cis cells have evolved some kind of molecular by-pass, allowing them to overcome the repair-inducing or lethal effects of the UV irradiation; or, the beginning of the apoptosis process is more delayed in them. Nevertheless, the low response of the cells to UV rays is probably connected to the higher resistance of Toledo-cis to cisplatin, when compared to the sensitive cell line. Pg-P activity has no effect on the development of cisplatin resistance in the cell lines described.

Our studies also presented how apoptosis was induced by the tested compounds in human ovarian cancer cells sensitive to cisplatin and resistant to cisplatin. The cytostatic activity of the 134 (**2**), 138 (**3**), and TCAP(**4**) compounds was investigated by an MTT reduction assay with exposure of cell cultures for 72 h. TCAP compounds is 60-times less toxic to normal lymphocytes than cisplatin, which is a favorable property in the context of testing potential anticancer agents. The results showed that ruthenium compounds 134 (**2**) and 138 (**3**) break partially or completely the cisplatin resistance by activating the caspase 8-dependent apoptosis pathway and loss of mitochondrial membrane potential. The cytotoxic effect of these compounds against the tested cells is marked already after several tens of minutes of exposure. Platinum compounds, cisplatin and TCAP(**4**), also have a cytostatic effect, but their action requires more time of exposure, i.e., several to several dozen hours. None of the compounds discussed shows any effect on topoisomerase activity in vitro.

Continuing the studies on the mechanisms underlying the cytotoxicity of potential drugs, we have described several aspects of anticancer activity of ruthenium(II) and platinum(II) complexes with aminoflavone bioligands. Further research in this area should focus on the limitations of current metal complexes and the development of novel compounds with increased specificity and efficacy along with reduced side-effects. Furthermore, developing approaches for overcoming resistance to platinum drugs is challenging due to the complexity of the resistance reaction. Therefore, it is important to recognize which factors contribute most to resistance in order to find ways to overcome the problem.

## Figures and Tables

**Table 1:** Summary of the IC_50_ values of the tested compounds across a panel of cancer cells and human lymphocytes.

IC_50_ (µM/L)					
	Cisplatin (1)	134 (**2**)	138 (**3**)	TCAP(**4**)	Ligand
A2780	0.2 ± 0.1	2.5 ± 0.2	1.8 ± 0.1	6.5 ± 0.3	>100
A2780cis	16.8 ± 1.5	4.6 ± 0.2	1.5 ± 0.4	15.1 ± 0.6	>100
Toledo	0.5 ± 0.07	0.5 ± 0.1	0.6 ± 0.04	8.8 ± 0.8	>100
Toledo-cis	8.3 ± 0.6	2.9 ± 0.2	2.8 ± 0.1	12.6 ± 1.2	>100
Lymphocytes	0.2 ± 0.1	3.4 ± 0.3	1.6 ± 0.1	11.9 ± 0.7	>100
**Resistance Factor Defined as IC_50_(Resistant)/IC_50_(Sensitive)**					
RF (IC_50_ A2780cis/IC_50_ A2780)	84	1.8	0.8	2.3	
RF (IC_50_ Toledo-cis/IC_50_ Toledo)	17	5.8	4.6	1.4	

## Data Availability

The experimental data presented in this study are available on request from the corresponding author.
